# Ethiopian teachers: their knowledge, attitude and practice towards epilepsy

**DOI:** 10.1186/s12883-016-0690-4

**Published:** 2016-09-08

**Authors:** Meron Awraris Gebrewold, Fikre Enquselassie, Redda Teklehaimanot, Seid Ali Gugssa

**Affiliations:** 1 Addis Ababa Univeristy, College of Health Sciences, School of Medicine, Department of Neurology, Addis Ababa, Ethiopia; 2 Addis Ababa University, College of Health Sciences, School of Public Health, Department of Statistics, Addis Ababa, Ethiopia

**Keywords:** Epilepsy, Ethiopia, KAP, Schoolteacher

## Abstract

**Background:**

In Ethiopia where the burden of epilepsy is highest among school age children and teenagers, and where people with epilepsy (PWE) and their relatives suffers from high level of perceived stigma, there had not been any study that assessed the knowledge, attitude and practice of teachers towards PWE. This study aims to assess and understand the social and demographic determinants of knowledge, attitude and practice of teachers towards PLW in Addis Ababa, Ethiopia.

**Methods:**

Multistage cluster sampling procedure was used to identify twenty schools from three sub cities of Addis Ababa, Ethiopia. Standardized self administered questionnaire was used to collect data from 845 volunteer teachers in the pre identified schools. Frequencies were used to characterize the demographic variables while multiple response frequencies were used to characterize the multiple response variable sets. Non-parametric statistical methods were used to describe the association among the demographic variables of interest and the count sums of multiple response variables which were grouped into biologically and culturally plausible responses.

**Results:**

The most common biologically plausible responses were: brain diseases (26.5 %) from causes, allow my offspring to play with PWE (19.1 %) from attitude, protect the subject from injury (20.4 %) from first aid measures and seek help from medical doctors (52.2 %) from epilepsy treatment. On the contrary, the most common culturally plausible responses were: psychiatric illness (12.9 %) from causes, epilepsy be cured before attendance to school (21.6 %) from attitude, smelling the smoke of struck match (14.2 %) from first aid measures and Holy water treatment (20.3 %) from epilepsy treatment suggestions. The biologically and culturally plausible responses were negatively correlated. Level of education was positively associated with biologically plausible responses while teaching experience was negatively correlated with culturally plausible responses.

**Conclusion:**

A high percentage of teachers in Addis Ababa considered epilepsy as a psychiatric illness closely linked to insanity. This explains their suggestions of Holy water treatment and Church healing sessions as epilepsy remedies. This is in agreement with Ethiopian culture, in which evil spirit and insanity are believed to be better treated by religious remedies than with modern medical treatments. Incorporating special needs educational training courses in the curriculum of teachers training may help them shift their knowledge, attitudes and practices from that of the culturally plausible to biologically plausible one.

## Background

Worldwide at least 50 million people live with epilepsy, and more than 80 % of them reside in developing countries [1]. Epilepsy stigma is especially prevalent in developing countries, and its dire social, psychological and economic consequences have become a major public health problem, magnifying the disability of epilepsy itself [2, 3].

For centuries religious and cultural taboos had influenced the type of care and treatment for people with epilepsy (PWE) [2]. In many African communities the myth that surrounds epilepsy continues to be an important cause of a wide epilepsy treatment gap. PLWE in these communities resort to complementary and alternative medicines and underutilize modern treatments [3, 4].

In Ethiopia 81 % of PWE and their relatives suffer from perceived stigma [5]. Students with epilepsy reportedly experience significantly higher level of stigma compared with other types of occupations [5]. According to a large community based Epidemiological study, the incidence and prevalence of epilepsy in Ethiopia were estimated to be 64/100,000/year and 5.2/1000, respectively [6, 7]. The highest age specific incidence occurred among the youngest age groups 0–9 and 10–19 years [6]. More than 85 % of PWE in Ethiopia do not receive epilepsy treatment. Ninety percent of the untreated were unaware of the existence of treatment for epilepsy, while only 4 % of them cited cost as a reason for not receiving treatment [8].

Ninety-seven percent of teachers working in Zambia were found to have high level of epilepsy awareness and majority (>70 %) of them recognized brain disorder as the commonest cause of epilepsy [9]. Evil spirit possession and witchcraft were considered as causes of epilepsy by 20 and 16.8 % of teachers respectively. Close to 30 % considered epilepsy as contagious disease and only 1.5 % of them as insanity [9]. In south East Asia 55–68.2 % of teachers did not provide first-aid to actively seizing students [10–12]. When they provide first-aid, they used potentially harmful interventions like inserting a spoon into the mouth (40.4 %), pouring animal excreta on the face of the subject (13.9 %) and having them smell leather shoes (15.7 %) [10].

To our knowledge this is the first KAP study in Ethiopian teachers. The objective of this study is to assess and understand the social and demographic determinants of knowledge, attitude and practice of teachers towards PWE in Addis Ababa, Ethiopia. The findings of this study would hopefully serve as a stepping stone for future large scale community based educational intervention programs that focus on teachers. This is especially true for Ethiopia where the burden of epilepsy is highest among school age children and teenagers, and where there is improving nationwide access to modern education.

## Methods

### Study overview and experimental design

#### Study setting

Addis Ababa, the capital city of Ethiopia, has ten sub-cities. The education system in the capital is divided into primary (1–8th grade) and secondary [junior high (9–10th) and senior high (11–12th grade)] levels. Seventy one percent of the schools were owned by heterogeneous nongovernmental owners that included private, local community, foreign community, religious community and other organizations. In the year 2012–2013, one thousand and forty schools were registered under Addis Ababa City Administration Education Bureau. Eighty percent of the schools were primary schools and the remaining 20 % were secondary schools [13].

#### General procedures, recruitment, and data collection

##### Eligibility and sampling procedure

**Fig. 1:**
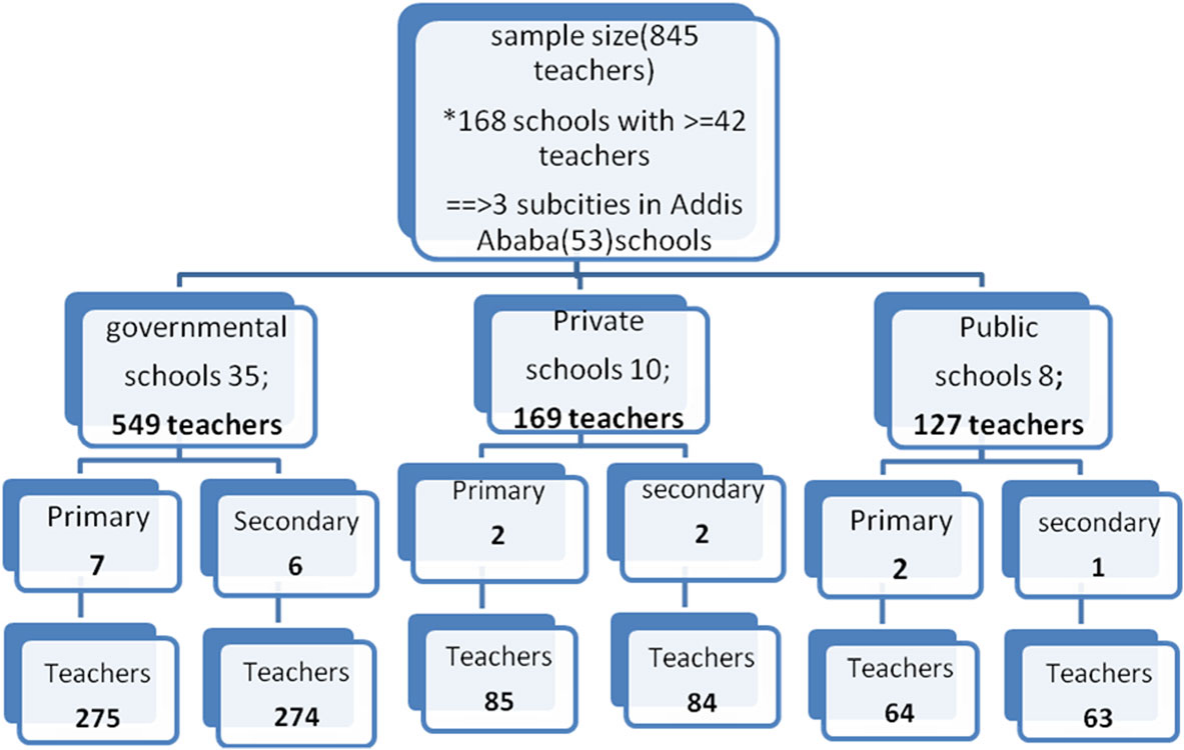
Multistage Cluster sampling of teachers from three Sub-Cities of Addis Ababa Ethiopia, 2013

For logistical reasons government, private and public owned schools that had greater than or equal to 42 teachers were considered eligible to be included in the study. To minimize the design effect of cluster sampling, the sample size calculated from Z value of 1.96, epilepsy awareness of 50 % and an error of estimate of 0.05 had been doubled. The corrected sample size, eight hundred and forty-five, was then divided by the minimum desired number of teachers in a given school, forty-two, to determine the number of school clusters, twenty. Multistage cluster sampling with three sampling units: Sub-city, type of school ownership and the level of the school were used as shown in Fig. 1 to identify the cluster unit schools from the list of schools obtained from Addis Ababa City Administration Education Bureau. Accordingly, from 3 Sub-Cities 275 teachers from 7 Governmental Primary schools, 274 teachers from 6 Secondary Governmental schools, 85 teachers from 2 Primary Private schools, 84 teachers from 2 Secondary Private schools, 64 teachers from 2 Primary Public schools and 63 teachers from 1 Secondary Public school were randomly selected.

##### Survey instrument

The questionnaire had two parts: The first part of the instrument contained identification and general demographic data such as age, gender, ethnicity, religion, educational status, and marital status, number of children, level of school taught, and years of experience as a teacher.

The second and the main body of the instrument had three sub-categories: knowledge, attitude and Practice. The Knowledge section assessed: epilepsy awareness, causes and manifestations. The Attitude section probed what teachers think the relationship should be like among epileptic and non-epileptic students, among epileptic students and their family, and the relationship teacher would like to have in a class room with their epileptic students. The Practice section assessed what teachers would do as a first aid measure to a student with active seizure and where they would recommend an epileptic student be treated. In order to maintain the standard of the survey material other similar studies were identified and relevant questions were selected and modified to make them appropriate to the local cultural. The selected questions were first forward translated into Amharic, the official language of the country, and then back translated into English using the standard translation procedure. The translated questionnaire was then pre-tested on 15 randomly selected school teachers in Addis Ababa which had helped to further redirect and rephrase some of the questions.

##### Ethical considerations, data collection and safety

The Institutional Review Board of the College of Health Sciences of Addis Ababa University approved the study to be conducted at the pre-identified schools in Addis Ababa, Ethiopia. Data was collected from August 1 to August 30, 2013 using the pretested self-administered questionnaire from all teachers who had consented to participate in the study at the selected schools. At the end of the data collection procedure the study participants were given health education about epilepsy. Each questionnaire was checked for completeness, assigned a code, kept in a secured file, cleaned and entered in password protected electronic database.

##### Exclusion criteria

After preliminary assessment of the data, teachers with history of epilepsy were excluded from the final analysis to avoid bias.

##### Study variables and operational definitions

The demographic variables listed above were used as explanatory variables for the count sums of the knowledge, attitude and practice (KAP) outcome variables. The count sums of the outcome variables were grouped into biologically and culturally plausible responses.

The biologically plausible variables were those variables for which the respondents had ticked ‘Yes’ to one or more of the following causes, manifestations, attitudes, first aid measures and treatment recommendations: Genetic disorders, trauma to the head, brain infection, brain tumor, and brain disease from causes; convulsions, drooling of saliva with loss of consciousness, blank staring, transient change in behavior and brief period of forgetfulness from manifestations; allow PWE in my class, PWE have normal intelligence, epilepsy could be controlled, allow my child to play with PWE, allow my child to marry to PLWE from attitude; Calling the school nurse/doctor for help when available, placing the child on his/her side, clearing airway, provide mouth to mouth breathing if child is not breathing, removing the child from a potentially dangerous area from first aid measures; treatment by doctor or surgeon from treatment recommendation were considered as biologically plausible responses.

The culturally plausible variables were also grouped into categories of causes, manifestations, attitudes, first aid measures and treatment recommendations. In this context causes included curse from God, possession by evil spirit, witchcraft, psychiatric illness. Manifestations included higher incidence of insanity in PWE, and epilepsy is contagious. Attitudes included PWE should be placed in special class and epilepsy should be cured before coming to school. First aid measures included insert spoon/gag into the mouth, pour water on the face, smell a struck match, and put sweets into the mouth of the seizing patient. Treatment recommendations included over-the-counter medications, help from Traditional healers, wearing an amulet, Church healing sessions, Holy water, and reciting the Holy Quran.

### Data analysis and statistical methods

Data was analyzed using SPSS/PC version 20.0, software packages for statistical analysis IBM Company/International Business Machines/IBM. Com, U.S.A. The demographic characteristics of the teachers were categorized and frequencies were calculated. Data was explored for skewness and kurtosis at *p* > 0.05. The homogeneity of variance was verified using the non-parametric Levene’s test at *P* > 0.05.

Where multiple responses were allowed the variable sets were defined before calculating the percent of responses and percent of cases for each predefined multiple response sets. Each variable response was categorized to either biologically or culturally plausible as detailed in the operational definition. These response variables were nominal variables with a response that is coded as ‘Yes’ or ‘No’ (‘1’ and ‘0’) respectively. Those who responded ‘Yes’ to biologically and culturally plausible set of variables were counted and summed separately to calculate the biologically plausible and culturally plausible Knowledge, Attitude and Practice (KAP) sum scores.

The biologically and culturally plausible KAP scores were assessed for correlation using the spearman’s correlation at *P* < 0.05. Likewise the correlation among the KAP scores and continuous demographic variables (age, calculated age at employment, years of experience and number of children) were determined using the Spearman’s correlation coefficient at *P* < 0.05.

The Kruskal- Wallis analysis of variance with post hoc testing was performed to determine the association among the KAP scores and categorical demographic variables (gender, educational status, level of school taught, ethnicity and religion).

## Results

A total of 844 teachers consented to participate in the study. Twenty four teachers (2.8 %) were excluded from the final analysis as they had a history of epilepsy. Majority of the respondents were single (55.7 %), Ethiopian Orthodox Christian (80.6 %), males (58.1 %), Amhara by ethnicity (54.1 %), taught at primary schools (50.2 %), and had attained diploma or college degree level of education (90 %). The remaining 10 % of teachers had school leaving certificate (5.5 %), certificate from Teachers Training Institute (1.3 %) or master’s degree (3.3 %). The median age of the teachers was 29 years (inter-quartile range 25–37 years). The median teaching experience was 6 years (inter-quartile range 3–13 years), and had a median of 2 children (inter-quartile range 1–3 children).

The median KAP score for the biologically and culturally plausible responses were 9.0 and 6.5 respectively. The inter quartile range for the biologically and culturally plausible KAP scores were 4 and 3 Table 1.

**Table 1:** Summary statistics of teachers KAP scores for biologically and culturally plausible responses in Addis Ababa, Ethiopia, 2013

	Range	Minimum	Maximum	Percentiles			Mean	Std. Deviation
				25	50	75		
Teachers with Biologically plausible responses	18	0	18	7.0	9.0	11.0	8.8104	3.17719
Teachers with culturally plausible responses	18	1	19	5.0	6.5	8.0	6.6327	2.12876

Ninety percent of the teachers knew epilepsy as a disease and the most common source of information was acquaintances with PWE (51.3 %), followed by public media (36.9 %) and medical doctors (2.3 %) (Table 2). Among those who had acquaintances with PWE, had come across PWE in person (67.2 %), and had a student with epilepsy in class (28.6 %) accounted for 95.8 % of the responses to personal acquaintances with PWE (Table 3).

**Table 2:** Knowledge of teachers about epilepsy as a disease and their source of information in Addis Ababa, Ethiopia, 2013

	Frequency	Percent	Cumulative Percent
Do not know about epilepsy	78	9.5	9.5
Acquainted with someone with epilepsy	420	51.3	60.8
Heard from Public media	303	36.9	97.7
Learnt from Doctors	19	2.3	100.0
Total	820	100.0

**Table 3:** Personal acquaintances of teachers to People with epilepsy (PWE) in Addis Ababa, Ethiopia, 2013. Y

	Responses		Percent of cases
	N	Percent	
Relative of mine had epilepsy	46	4.1 %	6.0 %
Know a PWE in person	749	67.2 %	98.0 %
Had students with Epilepsy in class	319	28.6 %	41.8 %
Total	1114	100.0 %	145.8 %

Y = multiple response allowed

The three most common biologically plausible responses concerning the causes of epilepsy were brain disease (26.5 %), head trauma (18.4 %) and genetic disorder (10.0 %). On the contrary psychiatric illness (12.9 %), evil spirit (6.4 %) and witchcraft (1.8 %) were the most common responses from the culturally plausible causes of epilepsy (Table 4). Teachers recognized convulsive forms more easily than non-convulsive forms of epilepsy (Table 5).

**Table 4:** Responses of teachers to the causes of epilepsy in Addis Ababa, Ethiopia, 2013. Y

	Responses		Percent of cases
	N	Percent	
Genetic Disorder	169	9.9 %	20.0 %
Head Trauma	313	18.4 %	37.1 %
Brain Infection	74	4.4 %	8.8 %
Brain Tumor	151	8.9 %	17.9 %
Brain Disease	451	26.5 %	53.4 %
Psychiatric illness	219	12.9 %	25.9 %
Curse of God	9	0.5 %	1.1 %
Evil Sprit	109	6.4 %	12.9 %
Witchcraft	31	1.8 %	3.7 %
Not Sure	173	10.2 %	20.5 %
Total	1699	100.0 %	201.3 %

Y = multiple response allowed

**Table 5:** Responses of teachers to the manifestations of epilepsy in Addis Ababa, Ethiopia, 2013. Y

	Responses		Percent of Cases
	N	Percent	
Convulsion	705	32.7 %	83.5 %
Loss of consciousness with Drooling of saliva	608	28.2 %	72.0 %
Brief Behavioral change	197	9.1 %	23.3 %
Blank staring	252	11.7 %	29.9 %
Brief period of forgetfulness	361	16.7 %	42.8 %
Not Sure	36	1.7 %	4.3 %
Total	2159	100.0 %	255.8 %

Y = multiple response allowed

The most common non-stigmatizing responses were: Epilepsy could be cured or controlled (14.8 %) allow my offspring to play with PWE (19.1 %), and allow my child to marry PWE (7.5 %). Although 89.2 % of the teachers would allow PWE into their class, the majority (76.7 %) of them preferred that the epilepsy be cured or controlled before attendance, accounting for 21.6 and 19.1 % of the responses respectively. PWE were perceived insane more than infectious explaining 9.4 and 0.2 % of the responses respectively (Table 6).

**Table 6:** Responses of teachers to questions that assessed their attitude towards PWE in Addis Ababa, Ethiopia, 2013. Y

	Responses		Percent Cases
	N	Percent	
Epilepsy is contagious	8	0.2 %	1.0 %
PWE have higher Insanity	320	9.4 %	38.9 %
Epilepsy could be cured or controlled	501	14.8 %	60.9 %
PWE have normal intelligence	176	5.2 %	21.4 %
Allow PWE in my class	734	21.6 %	89.2 %
Prefer to have PWE in my class after epilepsy is cured/controlled	631	18.6 %	76.7 %
Prefer PWE be in a separate class	125	3.7 %	15.2 %
Allow my offspring to play with PWE	647	19.1 %	78.6 %
Allow my child to marry a PWE	254	7.5 %	30.9 %
Total	3396	100.0 %	412.6 %

The most common biologically plausible first aid measure responses were: Protect from injury (20.4 %), clear the air ways (14.6 %), call a doctor for help (14.5 %) and place seizing students on their side (12.5 %). Smelling the smoke of struck match (14.2 %), pouring water on the face (7.8 %), and inserting a gag into the mouth of seizing subject (6.7 %) were the commonest culturally plausible first aid measure responses (Table 7).

**Table 7:** Responses of teachers the way they would provide first-aid to seizing student in Addis Ababa, Ethiopia, 2013. Y

	Response		Percent of cases
	N	Percent	
Place the student on her/his side	291	12.5 %	36.5 %
Will clear the air way	339	14.6 %	42.5 %
Will insert a spoon of gag into the mouth	156	6.7 %	19.5 %
Will provide mouth to mouth breathing	133	5.7 %	16.7 %
Will protect from injury	474	20.4 %	59.4 %
Make smell the smoke of a struck match	329	14.2 %	41.2 %
Will pour water on face of the subject	182	7.8 %	22.8 %
Will put sweets in the mouth	82	3.5 %	10.3 %
Will call Doctor or nurse	336	14.5 %	42.1 %
Total	2322	100.0 %	291.0 %

Y = multiple response allowed

Treatment by medical doctors (52.2 %) or by Surgeons (6 %) were the most common biologically plausible epilepsy treatment responses while Holy water treatment (20.3 %), Church healing sessions (11.0 %), and treatment by Traditional Healers (2.1 %) were the most common culturally plausible epilepsy treatment responses (Table 8).

**Table 8:** The response of teachers suggestion to epilepsy treatment in Addis Ababa, Ethiopia, 2013. Y

	Responses		Percent of cases
	N	Percent	
Treatment by medical doctor	742	52.2 %	87.9 %
Over the counter medicine	26	1.8 %	3.1 %
Treatment by surgeon	86	6.0 %	10.2 %
Treatment by Traditional healers	30	2.1 %	3.6 %
Church healing sessions	156	11.0 %	18.5 %
Recite The Holly Quran	37	2.6 %	4.4 %
Holly Water Treatment	288	20.3 %	34.1 %
Wearing Amulet	22	1.5 %	2.6 %
No need to treat	10	0.7 %	1.2 %
Not sure	24	1.7 %	2.8 %
Total	1422	100.0 %	168.5 %

Y = multiple response allowed

A Shapiro-Wilk’s test (*p* < 0.05) and an inspection of the skewness and kurtosis measures showed that the KAP scores were not approximately normally distributed. A non-parametric Levene’s test was used to verify the equality of variance in the samples (*p* > 0.05).

The Spearman’s correlation coefficient between biological and culturally plausible KAP scores was *r* = −0.236 with *p* < 0.01. There was significant negative correlation between culturally plausible KAP score and teaching experience (*r* = − 0.099, *P* < 0.004) and age at interview (*r* = − 0.118 and *p* < 0.001). The other continuous explanatory variables (calculated age at employment *r* = −0.036 and *P* = 0.292, and the number of children teachers had *r* = − 0.080 and *p* = 0.115) were not significantly correlated with culturally plausible KAP sum score. None of the continuous explanatory variables (age at interview *r* = 0.009 and *p* = 0.802, calculated age at employment *r* = 0.035 and *p* = 0.313, number of children teachers had *r* = 0.006 & *p* = 0.911, and teaching experience of teachers *r* = 0.002 and *p* = 0.953) were significantly correlated with the biologically plausible KAP score (Table 9).

**Table 9:** Spearman’s rho correlation coefficient by demographic characteristics of teachers for biologically and culturally plausible KAP sum of scores in Addis Ababa, Ethiopia, 2013

Demographic characteristics	Teachers with Biologically plausible responses		Teachers with culturally plausible responses	
	Correlation Coefficient	Sig. (2-tailed)	Correlation Coefficient	Sig. (2-tailed)
Age at interview	.009	.802	-.118	.001
Number of off springs	.006	.911	-.080	.115
Years of experience as a teacher	.002	.953	-.099	.004
Age at employment	.035	.313	-.036	.292
Biologically plausible responses	1.0	-	-.236	.000
culturally plausible responses	-.236	.000	1.0	-

The Kruskal–Wallis H test showed significant association between level of education and biologically plausible KAP score (X ^2^ = 12.03 and *p* < 0.007) but showed no association with culturally plausible KAP score (X^2^ = 3.71, *p* = 0.29). The overall effect size of educational level explaining the group difference in the biologically plausible KAP score was 1.4 %. However, there were significant differences among high school, college, and masters levels of education. The effect size of the differences was highest (5 %) between high school and master graduates. The rest of the categorical variables (gender, ethnicity, the level of school taught, and religion) showed no significant associations neither with biologically plausible nor to culturally plausible KAP scores (Table 10).

**Table 10:** Kruskal-Wallis H test by demographic characteristics of teachers for biologically and culturally plausible KAP sum of scores in Addis Ababa, Ethiopia 2013

Demographic Characteristics		Teachers with Biologically plausible responses			Teachers with culturally plausible responses		
		Mean KAP Rank	Chi-Square	Sig.	Mean KAP Rank	Chi-Square	Sig.
Educational status	School leaving certificate	309.32	12.037	.007	396.03	3.712	.294
	Diploma	407.43			399.94		
	Degrees	433.67			431.45		
	Masters	426.91			401.86		
Religion	Orthodox Christian	422.28	4.976	.290	423.38	3.382	.496
	Muslim	417.41			402.76		
	Catholic Christian	553.50			306.50		
	Protestant Christian	400.09			416.91		
	Other	504.38			490.36		
Ethnicity	Oromo	416.91	3.459	.484	414.42	.658	.956
	Tigre	424.81			398.89		
	Amhara	396.87			401.64		
	Southern Nations and Nationalities	374.74			406.76		
	Others	437.12			384.17		

## Discussion

Ninety percent of teachers in Addis Ababa recognize epilepsy as a disease which is comparable to that of Zambian study [9]. Public media and doctors played minor roles in creating awareness towards epilepsy, compared to findings in India and Thailand [10, 12].

Forty-two percent of the teachers had a student with epilepsy in their class. This was high compared to Egypt at 10.6 % [14], India at 12 % [10] and Nigeria at 23.2 % [15] but was comparable to the figure in Thailand at 34 % [12].

Disorder of the brain was recognized as the most common cause of epilepsy by 53.4 % of teachers. Yet 25 % and 12.6 % of them considered epilepsy to be caused by psychiatric illness and evil spirit, respectively. These latter causes of epilepsy were concordant with the attitude held by 38.9 % of teachers, that PWE are more likely to have insanity.

Many communities in Africa (35–57 %) consider epilepsy as a contagious disease [16–18], but only 1 % of the teachers in our study considered epilepsy as contagious. This was far lower than the percentage found in Zambian teachers (28.2 %), but comparable to percentages found in Istanbul, (2.3 %), India (4.9 %) and Egypt (1.6 %) [10, 14, 19]. Teachers in Addis allowed their offspring to play with and marry to PWE at similar rates to those of Zambia, India and Thailand [9, 10, 12]. Only 5 % of teachers in Addis Ababa considered PWE to have normal intelligence. This compares to attitudes of teachers elsewhere who believe PWE to be mentally retarded, notably in Egypt and Greece (53 %) [14, 20].

Sixty-eight percent of the first aid measure responses were biologically plausible but many also suggested potentially harmful interventions such as smelling a struck match (41.2 %), pouring water on the face (22.8 %), and inserting a spoon into the mouth (19.5 %). These practices were comparable to that found in southeast Asia where inserting a spoon into the mouth (40.4 %), pouring animal excreta on the face (13.9 %) and smelling of leather shoes (15.7 %) are common practices [10].

Sixty percent of teachers in Addis Ababa considered epilepsy as curable disease and 98 % would advise their epileptic relatives be treated by medical doctors or surgeons. But significant proportion of them (52 %) would also suggest Holy water and Church healing sessions as alternative treatment options. The high level of culturally plausible epilepsy treatment suggestions may be explained by the widely held belief among teachers that epilepsy is a psychiatric illness associated with evil spirits and insanity.

The biologically and culturally plausible KAP scores were negatively correlated as expected. Educational status was positively associated with biologically plausible KAP score while teaching experience was negatively correlated with culturally plausible KAP scores. The variability explained by educational status and teaching experience in both biologically and culturally plausible KAP scores were small at 1.5 and 9 % respectively.

## Conclusion

Teachers in Addis Ababa had comparable awareness, knowledge, attitudes and practices towards PWE as in other resource-poor countries. In addition significant proportion of teachers in Addis Ababa considered epilepsy as a psychiatric illness closely linked to insanity. This explains their suggestions of Holy water treatment and Church healing sessions as epilepsy remedies. This is in agreement with Ethiopian culture, in which evil spirit and insanity are believed to be better treated by religious remedies than with modern medical treatments.

It is therefore important to incorporate special needs educational training courses in the curriculum of teachers training. This will hopefully help them shift their knowledge, attitudes and practices from that of the culturally plausible to biologically plausible one. In addition, special needs education should be offered as refresher course to graduate teachers particularly to those with certificate and diploma level of training, and to those with little teaching experience.

In this study, educational status and teaching experience had small effect size in explaining variability within biologically and culturally plausible KAP scores. Moreover, KAP studies are known to be biased to socially desirable responses rather than to true behavioral responses. Supplemental qualitative studies such as key informant interview will therefore help bridge the gap inherent to such quantitative knowledge, attitudes and practices studies.

## Abbreviations

AAU: Addis Ababa University
KAP: Knowledge, attitude and practice
PI: Principal investigator
PWE: People with epilepsy
SPSS: Statistical Package for Social science Student
